# Objective Recognition of Tinnitus Location Using Electroencephalography Connectivity Features

**DOI:** 10.3389/fnins.2021.784721

**Published:** 2022-01-04

**Authors:** Zhaobo Li, Xinzui Wang, Weidong Shen, Shiming Yang, David Y. Zhao, Jimin Hu, Dawei Wang, Juan Liu, Haibing Xin, Yalun Zhang, Pengfei Li, Bing Zhang, Houyong Cai, Yueqing Liang, Xihua Li

**Affiliations:** ^1^Jihua Laboratory, Foshan, China; ^2^Suzhou Institute of Biomedical Engineering and Technology, Chinese Academy of Sciences, Suzhou, China; ^3^Department of Otolaryngology, Head and Neck Surgery, Chinese PLA General Hospital, Institute of Otolaryngology, Beijing, China; ^4^Better Life Medical, Suzhou, China; ^5^Jiangsu Testing and Inspection Institute for Medical Devices, Nanjing, China

**Keywords:** tinnitus location, objective recognition, resting-state EEG, connectivity features, deep learning algorithms

## Abstract

**Purpose:** Tinnitus is a common but obscure auditory disease to be studied. This study will determine whether the connectivity features in electroencephalography (EEG) signals can be used as the biomarkers for an efficient and fast diagnosis method for chronic tinnitus.

**Methods:** In this study, the resting-state EEG signals of tinnitus patients with different tinnitus locations were recorded. Four connectivity features [including the Phase-locking value (PLV), Phase lag index (PLI), Pearson correlation coefficient (PCC), and Transfer entropy (TE)] and two time-frequency domain features in the EEG signals were extracted, and four machine learning algorithms, included two support vector machine models (SVM), a multi-layer perception network (MLP) and a convolutional neural network (CNN), were used based on the selected features to classify different possible tinnitus sources.

**Results:** Classification accuracy was highest when the SVM algorithm or the MLP algorithm was applied to the PCC feature sets, achieving final average classification accuracies of 99.42 or 99.1%, respectively. And based on the PLV feature, the classification result was also particularly good. And MLP ran the fastest, with an average computing time of only 4.2 s, which was more suitable than other methods when a real-time diagnosis was required.

**Conclusion:** Connectivity features of the resting-state EEG signals could characterize the differentiation of tinnitus location. The connectivity features (PCC and PLV) were more suitable as the biomarkers for the objective diagnosing of tinnitus. And the results were helpful for clinicians in the initial diagnosis of tinnitus.

## Introduction

Tinnitus refers to patient’s perception of sound in the ear without any external sound or electrical stimulation. Clinically, tinnitus is divided into subjective and objective tinnitus, and most patients report subjective tinnitus (Smit et al., 2015). There are 5–15% of people in the world who have experienced tinnitus, among them, 1–3% of tinnitus patients’ everyday life is seriously affected and need medical treatment (Tunkel et al., 2014; Gallus et al., 2015). Because tinnitus is a subjective perception for patients, its clinical detection and treatment are significant challenges (Pan et al., 2009). A multidisciplinary European guideline for tinnitus points out uniformity in the assessment and treatment of adult patients with subjective tinnitus (Cima et al., 2019). However, for the initial diagnosis of tinnitus, an efficient and objective method for recognizing and diagnosing tinnitus is still urgently needed.

Tinnitus is a health condition, not a disease. It is a symptom of pathological neurological activity that manifests as an unwanted auditory hallucination (Henry et al., 2020), and the complex pathophysiologic mechanism of tinnitus is still poorly understood.

On a sensory level, tinnitus is assumed to be caused by cochlear damage, but many tinnitus patients have normal audiograms, that is, there are no direct signs of cochlear damage (Schaette and McAlpine, 2011; Eggermont and Roberts, 2012). And the severance of the auditory nerve does not eliminate the sensation of tinnitus, and tinnitus is also not associated with the excessive activity of the auditory nerve (Müller et al., 2003). In rat experiments, the degree of reorganization of the auditory cortex is related to the intensity of tinnitus (Engineer et al., 2011). Tinnitus may originate in the dorsal cochlear nucleus (DCN) (Krauss et al., 2016), which is the auditory brainstem. The pathological changes in the activity of spontaneous neurons in the auditory brainstem can drive the reorganization of the auditory cortex (Rauschecker et al., 2010). A survey of rock musicians who felt transient tinnitus after practicing in a loud band (∼120 dB SPL for ∼2 h), showed that the gamma frequency power of the right temporal cortex was significantly increased during the appearance of transient tinnitus (Schlee et al., 2011), and importantly, which was not correlated with the degree of hearing loss (Ohlemiller et al., 2011).

On a macroscopic level, tinnitus was related to abnormally oscillating brain activity patterns (Schoisswohl et al., 2021), and neuroimaging studies also showed that there was a hyperactive auditory cortex during tinnitus (Mirz et al., 1999; Cunnane, 2019). Weisz et al. (2005) found that there was a significant enhancement of delta (1–4 Hz) and gamma (40–90 Hz) frequency power and a concomitant reduction of alpha (8–12 Hz) activity in tinnitus patients, and these changes mainly occurred in the temporal area. This statement was very consistent with the thalamocortical dysrhythmia model (TCD) (Llinás et al., 2005; Vanneste et al., 2018). A decrease in alpha power was associated with an increase in cortical excitability (Sauseng et al., 2005; Klimesch et al., 2007; Romei et al., 2008), the alpha desynchronization observed in chronic tinnitus reflected the release of inhibition, and thus facilitated the synchronization of neuronal activity. The synchronization by loss of inhibition model (SLIM) also supported this view, which explained the enhanced synchronization of auditory activity by a reduction of cortical inhibition (Weisz et al., 2007). However, the changes in brain activity that accompany tinnitus were not limited to the auditory cortices, a global brain model of tinnitus pointed out the phase coupling between the anterior cingulate cortex (ACC) and the right frontal lobe and the phase coupling between the ACC and the right parietal lobe were related to tinnitus. At the same time, in participants with a shorter tinnitus history, the gamma network was mainly distributed in the left temporal cortex, while in participants with a longer tinnitus duration, the gamma network was more widely distributed throughout the cortex (Schlee et al., 2011). This also reminded us of the potential relationship between the global brain function network connection and tinnitus.

Schlee et al. (2008) showed the existence of a tinnitus network by using a magnetoencephalogram (MEG) and found a wide range of abnormal functional connections in the brain. When conducting brain network anatomy research, we should focus on the spatial information interaction between brain regions and nodes (Zhang X. et al., 2021). Different brain regions did not work independently of each other, instead, they were connected in various long-distance networks (Bressler and Menon, 2010). One study on tinnitus patients found aberrant functional connectivity within the default mode network (DMN) (Cai et al., 2019). Chronic tinnitus patients had abnormal functional connectivity networks originating from ACC to other brain regions that were associated with specific tinnitus characteristics (Chen et al., 2018). Therefore, the brain functional network information may be a potential biomarker for tinnitus. Though those existing studies have evidenced the abnormal functional network in tinnitus patients, whether functional connection information can be used as the biomarker for tinnitus is less probed in previously reported studies.

In past studies, the time-domain features (rhythm signals) and frequency-domain features (power spectral density, PSD) of EEG signals were usually used as biomarkers to distinguish tinnitus and non-tinnitus, but these features were not obvious, and the accuracy of the identification was as high as 87% (Wang et al., 2017; Vanneste et al., 2018). We hope that functional connectivity features can better reflect the pathological features of tinnitus, and it will greatly improve the accuracy of the identification of tinnitus by using functional connectivity features as the biomarker.

At the same time, among studies that reported the localization of tinnitus-related signals, in about half of tinnitus patients, the percept occurred in the middle of the head or both ears. For others, tinnitus was perceived to be mainly on the left side, and some patients even feel that their tinnitus came from outside of the head (Baguley et al., 2013). The tinnitus might be heard as unilateral or bilateral (Meikle and Taylor-Walsh, 1984), some studies pointed out unilateral tinnitus was more commonly associated with underlying disease processes than bilateral tinnitus (Chari and Limb, 2018). Therefore, determining the location of tinnitus was one of the most important steps for the treatment of tinnitus in the cortex (Lee et al., 2019), because this information determined the type or order of treatments that the patient was given (Herraiz, 2008), and which might also enable us to probe the neurophysiology of tinnitus in ways heretofore not considered (Searchfield et al., 2015). If we can identify tinnitus symptoms and determine the location of tinnitus by using functional connectivity features, it will be of great help for clinicians.

Our research aim is to find the optimal solution for the objective recognition of possible tinnitus sources. As far as we know, there is no reference for the selection of functional connectivity features in the field of tinnitus recognition. We considered three functional connectivity features that were widely used in the field of emotion recognition (Lee and Hsieh, 2014; Gao et al., 2020; Moon et al., 2020), including the PCC, PLV, and TE. At the same time, the PLI was also included by us as a connected feature. PLV and PLI can measure the phase synchronization between time series nodes, and PCC and TE can measure the correlation between time series nodes, both of which can represent the connection status of network functions. At the same time, we also selected the mean and standard deviation of the rhythm of the six bands of EEG, such as δ delta (1–3.5 Hz), θ theta (4–7.5 Hz), α alpha (8–12 Hz), β beta (12.5–30 Hz), low γ gamma (30.5–48 Hz) and high γ(52–90 Hz), as well as the PSD, as the comparison features. We selected four classification algorithms, included two SVM, a MLP, and a CNN, to combine with the extracted features.

## Materials and Methods

### Participants

Participants with chronic tinnitus were recruited from the Otolaryngology-Head and Neck Surgery Department of Chinese PLA General Hospital and other hospitals from October 2018 to July 2019. Participants without tinnitus and hearing loss were recruited from the general population.

All participants in the tinnitus group had signs of definitive tinnitus for at least 3 months, and they also had normal hearing on the conventional hearing tests. Normal hearing was defined as follows: (1) A pure tone audiometric (PTA) threshold of 25 dB hearing level (HL) or better for all octave frequencies from 250 to 8,000 Hz; (2) transient evoked otoacoustic emission (OAE) with a signal-to-noise ratio (SNR) > 5 dB and a distortion product OAE with an SNR > 3 dB on OAE tests; (3) a waves I-III inter-peak latency < 2.4 ms and a wave V latency < 6.2 ms on 90 dB nHL click-evoked auditory brainstem response (ABR) tests; and (4) a normal tympanic membrane on otoscopy (Ahn et al., 2017). It had been proposed that the people with tinnitus who showed normal audiograms could have hidden hearing loss defined as damage to the auditory nerve fibers (Gollnast et al., 2017; Tziridis et al., 2021). Despite these possibilities, all patients with tinnitus in this study showed normal latencies in waves I-III of the ABRs and normal OAEs, which usually indicated the integrity of the peripheral auditory nerves (Møller et al., 1981; Møller and Jannetta, 1982) and normal function of the cochlear hair cells (Kemp, 1978; Mills and Rubel, 1994). To reduce the possibility of including patients with hidden hearing loss, and to ensure the cognitive abilities between patients and healthy participants were comparable, we included subjects who (1) were between 20 and 50 years old; (2) had no current or previous history of vertigo, Meniere’s disease, noise exposure, hyperacusis, or psychiatric problems; (3) had no history of head trauma or central nervous system disease and no anxiety or depression; (4) were not exposed to ototoxic drugs; and (5) had no complex or poorly defined tinnitus. Additionally, all patients completed a tinnitus questionnaire, which included a visual analog scale (VAS) and the Tinnitus Handicap Inventory (THI) questionnaire.

Forty-two participants with valid EEG data were selected for the study, which included 10 healthy participants, 12 bilateral tinnitus patients, 8 right-sided tinnitus patients, and 12 left-sided tinnitus patients (Table 1). The difference between the age and sex or duration of all participants was analyzed using the between-group *t*∼test, and *p* > 0.05. There was not a significant difference between these groups in terms of age and sex or duration. All participants were informed about the background and purpose of this study, and they all gave written informed consent. This study complied with the ethical principles of the Declaration of Helsinki.

**TABLE 1:** Overview of the patients with tinnitus.

Number	Sex	Age (year)	Localization	Duration (患病时间)	Tinnitus frequency (kHz)
1	Female	21	Both ears	2 y	4
2	Male	25	Both ears	3 y	0.5
3	Male	23	Both ears	3 y	8
4	Male	43	Both ears	1 y 6 m	4
5	Male	50	Both ears	6 y	4
6	Male	40	Both ears	8 y	4
7	Male	32	Both ears	1 y	4
8	Male	29	Both ears	0 y 3 m	4
9	Female	25	Both ears	0 y 6 m	0.5
10	Female	31	Both ears	5 y	4
11	Female	36	Both ears	5 y	4
12	Female	48	Both ears	7 y	4
13	Female	37	Right ear	4 y	0.125
14	Female	44	Right ear	0 y 4 m	8
15	Female	50	Right ear	1 y	3
16	Male	28	Right ear	0 y 3 m	4
17	Female	45	Right ear	0 y 3 m	8
18	Male	42	Right ear	1 y	0.5
19	Male	30	Right ear	3 y	4
20	Female	44	Right ear	1 y	8
21	Male	31	Left ear	1 y	0.5
22	Female	31	Left ear	0 y 6 m	0.125
23	Female	45	Left ear	0 y 6 m	0.125
24	Male	37	Left ear	0 y 3 m	0.125
25	Male	42	Left ear	0 y 6 m	0.2
26	Male	25	Left ear	0 y 3 m	6
27	Female	38	Left ear	0 y 3 m	0.15
28	Female	25	Left ear	0 y 4 m	1
29	Female	46	Left ear	1 y	2.175
30	Female	45	Left ear	5 y	6
31	Male	48	Left ear	1 y	4
32	Female	28	Left ear	0 y 5 m	4

### Electroencephalography Recordings

The 64-channel Neuroscan device recorded the EEG signals. The electrode position was set according to the international 10–20 electrode distribution. The sampling rate was 1,000 Hz, and the impedances were kept below 10 kΩ. During the EEG recording, the participants were asked to stay still, awake, and close their eyes in a sound and electrically shielded room. Each participant’s resting-state EEG signals were recorded for an estimated 10 min. According to reports, the global connectional properties in the brain stabilize with acquisition times as little as 5 min (van Dijk et al., 2010). And to ensure the availability of data, after acquiring the EEG data, the subject was asked whether he\she was sleepy during the resting state.

### Data Preprocessing

We used MATLAB-EEGLAB v14.1.2 toolbox for preprocessing. First, we performed interpolation processing on the insufficient lead data and deleted abnormally fluctuating time-period signals. Then, EEG signals were filtered by 50 Hz power frequency and bandpass-filtered to 0.5–90 Hz. Some research methods directly performed 0.5–48 hz band-pass filtering and ignored the beneficial components in the high-frequency signal, but the high-frequency γ rhythm (52–90 hz) was also related to tinnitus (Pierzycki et al., 2016). Next, baseline correction and REST re-reference of EEG signals (Yao, 2001) were performed. Last, the interference signals in EEG signals include ocular electricity, electrocardiogram (ECG), and other artifacts were filtered out by independent component analysis (ICA). The preprocessed signals were segmented at an interval of 10 s to expand the sample capacity.

**FIGURE 1:**
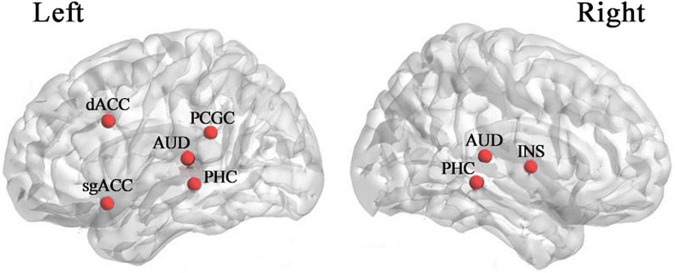
Cortical areas are associated with tinnitus (Vanneste et al., 2018). dACC dorsal anterior cingulate cortex, sgACC subgenual anterior cingulate cortex, PCGC posterior cingulate cortex, AUD auditory cortex, PHC parahippocampus, INS insula.

**FIGURE 2:**
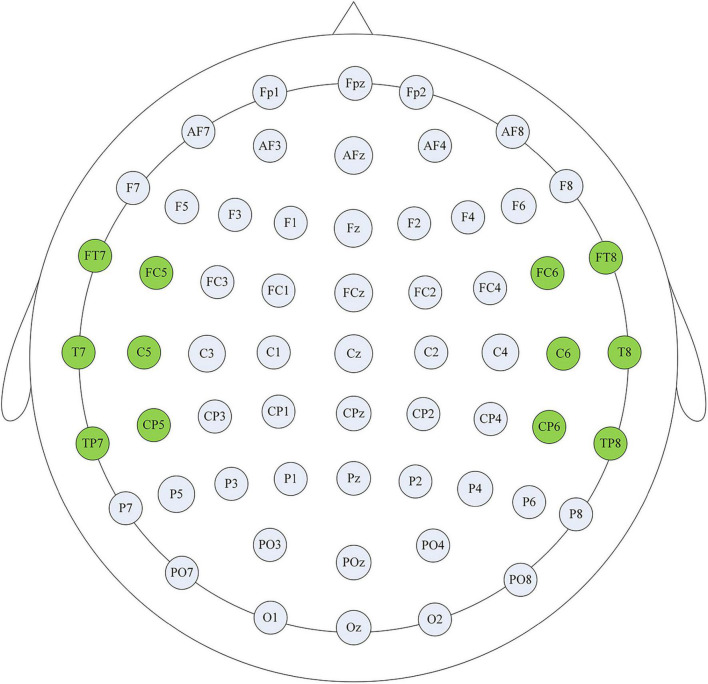
Channel selection display. The highlighted channels cover the auditory cortex on the left and right sides of the brain, and these channel data were used for subsequent analysis to improve the efficiency of clinical diagnosis.

Thalamocortical dysrhythmia (TCD) was a model proposed to explain divergent neurological disorders (Vanneste et al., 2018), which utilized the resting-state EEG signals of the left and right auditory cortex (AUD) areas to perform oscillation analysis of tinnitus disorders (Figure 1), including the parahippocampus (PHC), the dorsal anterior cingulate cortex (dACC), the subgenual anterior cingulate cortex (sgACC), the posterior cingulate cortex (PCGC) and the right insula (rINS). Imaging studies in patients with tinnitus had shown functional and structural abnormalities distributed in the auditory areas of the brain, including the cingulate cortex, PHC, and INS, and there was an increase in the activation of ACC and INS (Elgoyhen et al., 2015). Therefore, this study also selected the channel data of the auditory cortex on the left and right sides of the brain to be included in the next calculation and analysis, as shown in Figure 2, the channels including FT7, FC5, C5, T7, TP7, CP5, FC6, FT8, C6, T8, CP6, TP8. The efficiency of data processing can be greatly improved by reducing the calculation of channel data, and this targeted reduction will not make us lose important reference information. In the future, we may apply this research method to clinical diagnosis. As we all know, the 12-channel EEG acquisition experiment is easier than the 64-channel acquisition experiment, and the simplified method can also improve the efficiency of clinical diagnosis.

### Connectivity Measures

Four different connectivity measures were used in this study: PLV, PLI, PCC, and TE, which denoted the connection characteristics between different EEG channel signals. This study also considered the time-frequency domain features, including the rhythm and PSD.

#### Phase Locking Value

The PLV (Lachaux et al., 1999) denotes the phase synchronization between two signals, which is calculated as the average of the absolute phase difference. The value range of PLV is from 0 to 1, indicating perfectly independent or perfectly synchronization of the two signals, respectively. PLV is also an undirected connectivity feature.

$$P\,L\,V\,\left(i,k\right)=\frac{1}{T}\,\left|\sum_{t=1}^{T}\left(\varphi_{i}^{t}-\varphi_{k}^{t}\right)\right|$$

Where φ*^t^* is the phase of the signal at time *t*.

#### Phase Lag Index

PLI (Stam et al., 2007) is also a measure of phase synchronization. PLI between the signals is defined as follows:

$$PLI\left(i,k\right)=\frac{1}{T}\sum_{t=1}^{T}sgn\left(Im\left(Z_{i}\left(t\right)Z_{k}\left(t\right)*\right)\right)$$

where *Z*_*i*_(*t*) and *Z*_*k*_(*t*) for t = 1, …, *T* is the time-frequency representations of *i* and *k* signal in a given frequency band, note that *Z_i_* and *Z_k_* are complex-valued, *Sgn* denotes the sign function and the superscript, * denotes complex conjugation.

#### Pearson Correlation Coefficient

The PCC measures the linear relationship between two signals as a continuous number in [−1,1]. PCC values of −1 and 1 correspond to perfectly negative and positive linear relationships, respectively, and a PCC value of zero indicates that the two signals are not correlated. Let $x_{i}\,\left\{x_{i}^{1},x_{i}^{2},\ldots \,\ldots ,x_{i}^{T}\right\}$ indicate an EEG signal of the ith electrode, where *T* is the time length of the signal. The PCC of two signals *x_i_* and *x_k_*is calculated as:

$$P\,C\,C\,\left(i,k\right)=\frac{\frac{1}{T}\,\sum_{t=1}^{T}\left[\left(x_{i}^{t}-\mu_{i}\right)*\left(x_{k}^{t}-\mu_{k}\right)\right]}{\sigma_{i}*\sigma_{k}}$$

where μ and σ are the mean and standard deviation of the signal, respectively.

#### Transfer Entropy

The TE (Schreiber, 2000) describes the directed flow of information from a signal *x_i_* to another signal *x_k_*:

TE(i→k)

$$=\frac{1}{T-1}\,\sum_{t=1}^{T-1}\left[p\,\left(x_{i}^{t},x_{k}^{t},x_{k}^{t+1}\right)*\log_{2}\left(\frac{p\left(x_{k}^{t+1}|x_{i}^{t},x_{k}^{t}\right)}{p\left(x_{k}^{t+1}|x_{k}^{t}\right)}\right)\right]$$

A TE value of zero indicates that there is no causal relationship between the two signals, and TE belongs to the directed connectivity feature.

### Classification Methods

We selected four different machine learning algorithms to avoid the classification differences caused by the algorithm itself. The first two were the support vector machines (SVM) with a linear kernel function and two different cross-validation methods to improve the classification accuracy of the model, one was leave-one-out cross-validation (Loo-CV), and the other was 10-fold cross-validation (10-CV); The third was a multi-layer perception network (MLP), with parameter settings: the optimizer was “adam”, the alpha was 10^–7^, the hidden layer nodes were 200 for each of the two layers, the maximum number of iterations was 380, the initial learning rate was 0.001, and other parameters were the default parameter settings; The fourth was convolutional neural network (CNN) with long short-term memory model (CNN-LSTM), the layer details and parameters used for the CNN-LSTM model were shown in Table 2. All classification algorithms developed in the open-source Python libraries Scikit-Learn and TensorFlow.

**TABLE 2:** CNN-LSTM model architecture.

Layers	Types	Activation function	Output shape	Size of kernels	Filters/units	Stride	Rate of dropout
0	Input	–	1 × 66	–	–	–	–
1	1DCNN	Relu	1 × 16	3 × 1	16	1	–
2	1DCNN	Relu	1 × 32	3 × 1	32	1	–
3	LSTM	Tanh	1 × 32	–	32	–	–
4	LSTM	Tanh	32	–	32	–	–
5	Batch normalization	–	32	–	–	–	–
6	Flatten	–	32	–	–	–	–
7	Dense	Relu	16	–	16	–	–
8	Dropout	–	16	–	–	–	0.2
9	Dense	Relu	8	–	8	–	–
10	Dropout	–	8	–	–	–	0.2

In this study, a total of 2,312 valid segment data were calculated (including data from the healthy control group and three types of tinnitus patients), and the category calibration was completed for each valid segment, 1,622 groups were randomly selected as the training set, and 690 groups as the validation set. Each group of classification tests was repeated 10 times, and the results were the average of the classification accuracy and model computing time, respectively. The computer configuration: CPU Core I5 8th generation processor, GPU AMD R620, the highest frequency 1.8 GHz, and the running memory 16 GB. It is necessary to explain that the difference in calculator configuration will bring about differences in computing power and model computing time.

### Statistical Analysis

**FIGURE 3:**
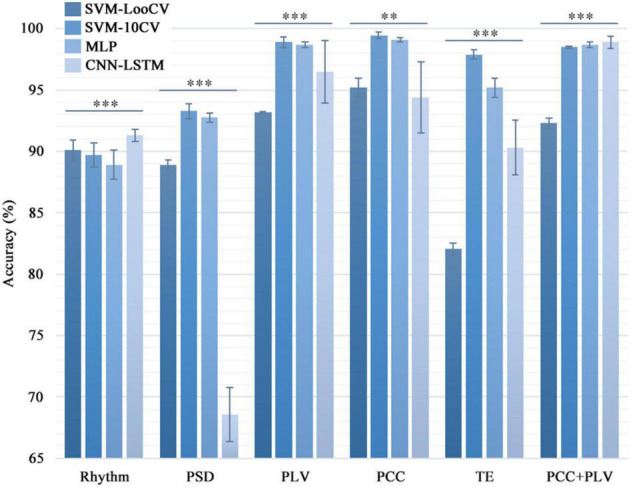
Comparison of the classification accuracy in different combinations of classifiers and features. Four machine learning algorithms were used to calculate the six feature data sets one by one to distinguish the healthy group and the three types of tinnitus patients. For the same feature set, the classification accuracy of the four models were significantly different (*p < 0.05, ^**^p < 0.01, ^***^p < 0.005). And the standard deviation showed that the SVM and MLP models were more stable than the CNN model.

The one-factorial ANOVAs and the Kruskal-Wallis ANOVAs (non-parametric) methods were used in the statistical analysis in this article. We used the one-factorial ANOVAs to analyze the recognition accuracy of the same feature combined with different machine learning algorithms (Figure 3) which could test whether there were significant differences between different classification methods. At the same time, the Kruskal-Wallis ANOVAs (non-parametric) were utilized to evaluate the calculation time for different approaches.

**FIGURE 4:**
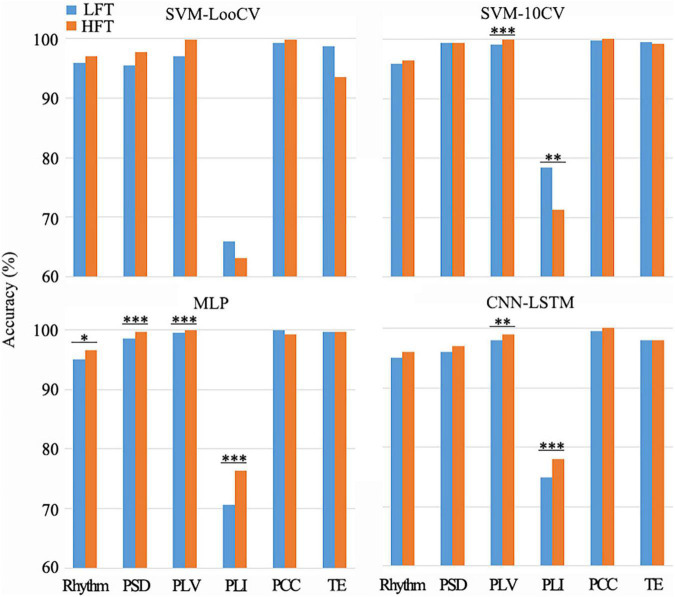
Differences between LFT and HFT in left-sided tinnitus patients in the combination of different methods. There were some combinations that showed a significant difference between LFT and HFT (*p < 0.05, **p < 0.01, ***p < 0.005). Among the groups that showed significant differences (*p* < 0.05), only when the SVM-10CV combined with PLI, the recognition accuracy of HFT was lower than LFT. And the other groups were all HFT with better recognition results.

It is known from the literature that low-frequency tinnitus (LFT) is perceived and processed differently from high-frequency tinnitus (HFT) (Zhang et al., 2020). However, in the tinnitus participants recruited in this study, we did not deliberately distinguish between patients with LFT or HFT. Among the tinnitus participants recruited in this study, there were two LFT patients and ten HFT patients in the bilateral tinnitus group; seven LFT patients and five HFT patients in the left-sided tinnitus group; and two LFT patients and five HFT patients in the right-sided tinnitus group. To explore whether the EEG data of patients with LFT or HFT has an impact on the diagnostic methods of this study, we selected the left-sided tinnitus group with an almost balanced LFT and HFT for testing. We combined the LFT and HFT in the left-sided tinnitus group with the data of the same healthy control group into a new data set and then used the method in this study to classify tinnitus disorders. Next, we used the one-factorial ANOVAs method to evaluate the difference between the LFT and HFT during using different combinations of methods (Figure 4).

**FIGURE 5:**
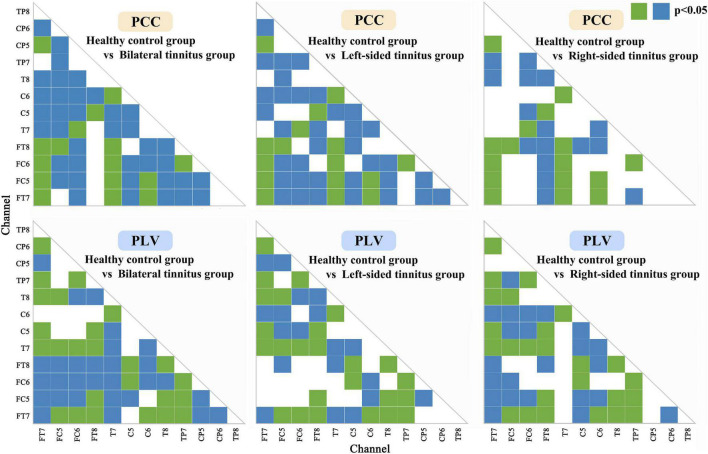
Statistical analysis of Phase lock value and Pearson correlation coefficient matrix. Both the green block and the blue block indicated that there were significant differences (*p* < 0.05) in the feature data between the healthy control group and the tinnitus group in this area, and the green block was the significant difference area that both existed in the three tinnitus groups.

We also did a statistical analysis of the two connectivity features PCC and PLV with excellent recognition accuracy, and the results are shown in Figure 5. Both the green block and the blue block indicated that there were significant differences in the feature data between the healthy control group and the tinnitus group in this area, and the green block was the difference area that both existed in the three tinnitus groups.

## Results

### Results for Classification

Table 3 shows the results of the SVM-LooCV, SVM-10CV, MLP, CNN-LSTM algorithms when classifying data based on Rhythm, PSD, PLI, PLV, PCC, TE features alone or the combination of PCC and PLV features. Figure 3 shows classification accuracy using four methods with different features (PLI is not mentioned here because its identification result is lower than other features). As can be seen from Table 3, classification accuracy is better when using connectivity features, especially PLV, PCC, and TE. The SVM-10CV classifier based on the PCC feature shows the highest classification accuracy (99.42%), and included the PLV feature achieves an accuracy of 98.9%, at the same time, the MLP classifier based on the PCC feature achieves an accuracy of 99.1% and included the PLV feature achieves an accuracy of 98.7%. These four combinations of classifier and alone feature are the best result so far.

**TABLE 3:** Classification accuracy and computing time using four methods with different features.

Features	SVM-LooCV		SVM-10CV		MLP		CNN-LSTM	
	Accuracy	Times	Accuracy	Times	Accuracy	Times	Accuracy	Times
Rhythm	90.09	480	89.7	420	88.9	4.6	91.3	502
PSD	88.88	540	93.27	150	92.76	3.5	68.5	533
PLV	93.16	366	98.9	142	98.7	3	96.5	474
PLI	45.65	600	54.7	220	52.9	6.8	43	570
PCC	95.24	300	99.42	104	99.1	3	94.4	578
TE	82.09	600	97.9	390	95.2	5.2	90.3	461
PLV+PCC	92.3	800	98.5	120	98.7	3.3	98.9	470

At the same time, the statistical analysis of the calculation results shows that there are significant differences in the recognition accuracy of different classification models (*p* < 0.01). The standard deviation can show that the SVM and MLP models are more stable than the CNN model. The possible reason is that CNN needs a larger sample size to make the experimental results more accurate and stable.

Comparing the classification results from Table 3 and Figure 3, it is shown that the use of the PLV or PCC features results in about 6.5% higher classification accuracy than the PSD feature, regardless of the classification algorithm, and the difference is even greater than Rhythm feature. Thus, PLV and PCC show to be more suitable than other features when used to classify tinnitus.

In addition to the classification accuracy, there is another important factor for judging the quality of a classifier, which is computing time. As can be seen from Table 3, there are significant differences in the average calculation time of the four model algorithms (*p* < 0.001). The computing time of SVM-LooCV and CNN-LSTM is similar, and they are the longest in the four classifiers. Compared with them, the computing efficiency of SVM-10CV is improved by 2.4 times. And MLP runs the fastest, with an average computing time of only 4.2 s, which is 52 times that of CNN-LSTM and 120 times that of SVM. Thus, MLP is more suitable than other methods when a real-time classification is required.

There was a difference in the recognition accuracy between LFT patients and HFT patients, as shown in Figure 4. Among the groups that showed significant differences (*p* < 0.05), only when the SVM-10CV combined with PLI, the recognition accuracy of HFT patients was lower than LFT patients. And the other groups were all HFT patients with better recognition results, which were 1.92% higher on average. The MLP classifier based on the PLV or PCC feature had the best comprehensive performance in this article. The MLP classifier combined with PLV features had very significant differences in the classification results of LFT and HFT, and the classification accuracy of HFT was 0.54% higher than that of LFT. Although the average classification accuracy of LFT patients was higher than that of HFT patients, there was no significant difference between them during the MLP classifier based on the PCC feature. In summary, the impact of LFT and HFT in this method was very small, and would not affect the identification of the location of tinnitus with low-frequency and high-frequency. However, this did not mean that we denied the difference between LFT and HFT, and it was also worthy of attention.

The calculation idea of SVM is to find the most suitable “linear segmenter,” also called hyperplane, for the input data set. This process generally needs to project the data set into a high-dimensional space, and the kernel function keeps working to find a hyperplane that can converge. It is more suitable for two classification problems, but for multi-class classification problems, it takes a long time. The calculation idea of the MLP network is to map a set of input vectors to a set of output vectors. It is a process of continuous simplification through the calculation of non-linear activation functions, so the calculation speed of the MLP is the fastest. In addition, it also has a back-propagation mechanism, which can continuously optimize the weight coefficients, and ultimately make the prediction error smaller and smaller. CNN is more commonly used in the classification of two-dimensional data, such as image data. However, the EEG data this time is one-dimensional data, which does not fully utilize the operating capabilities of the model. This may also be the reason for the poor performance of the model. During the analysis of the connectivity feature matrix, we assume that if the two-dimensional data of the matrix is used as the feature input in the future, it is likely to improve the classification accuracy and calculation efficiency of tinnitus again.

### Relationship of Connectivity Features and Tinnitus

**FIGURE 6:**
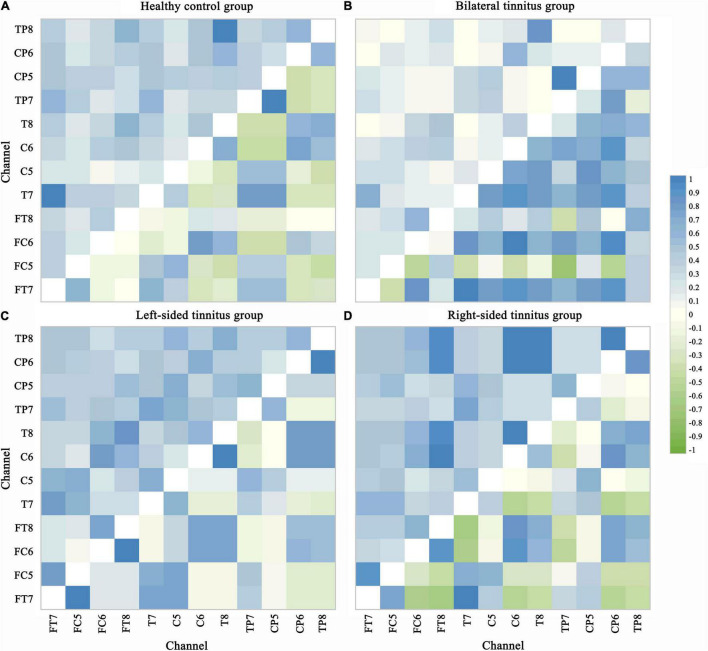
Phase lock value and Pearson correlation coefficient matrix. PLV and PCC are both undirected connection characteristics, there is only one value between the two channels. The upper left area represents the PLV matrices and the lower right area represents the PCC matrices in each picture. Visual inspection shows clear differences in the PLV matrices and PCC matrices. The value range of PLV is from 0 to 1, indicating perfectly independent or perfectly synchronization of the two signals, respectively. PCC values of –1 and 1 correspond to perfectly negative and positive linear relationships, respectively, and a PCC value of 0 indicates that the two signals are not correlated. **(A)** Healthy control group. **(B)** Bilateral tinnitus group. **(C)** Left-sided tinnitus group. **(D)** Right-sided tinnitus group.

In this paper, the classification accuracy of features PLV and PCC are excellent and stable. Therefore the values of PLV and PCC between each of the 12 selected channels are used to construct functional connection matrices for subsequent analysis. Since PLV and PCC are both undirected connection characteristics, there is only one value between the two channels, so in Figure 6, the upper left area represents the PLV matrices, and the lower right area represents the PCC matrices. Visual inspection shows clear differences in the PLV matrices and PCC matrices.

In Figure 5, the significant differences in most regions show the effectiveness of the two connectivity features (PLV and PCC) as biomarkers for tinnitus. In addition to areas with significant differences in common, there are many areas with significant differences in a single disease. Which also improves the classification accuracy of different localizations of tinnitus.

**FIGURE 7:**
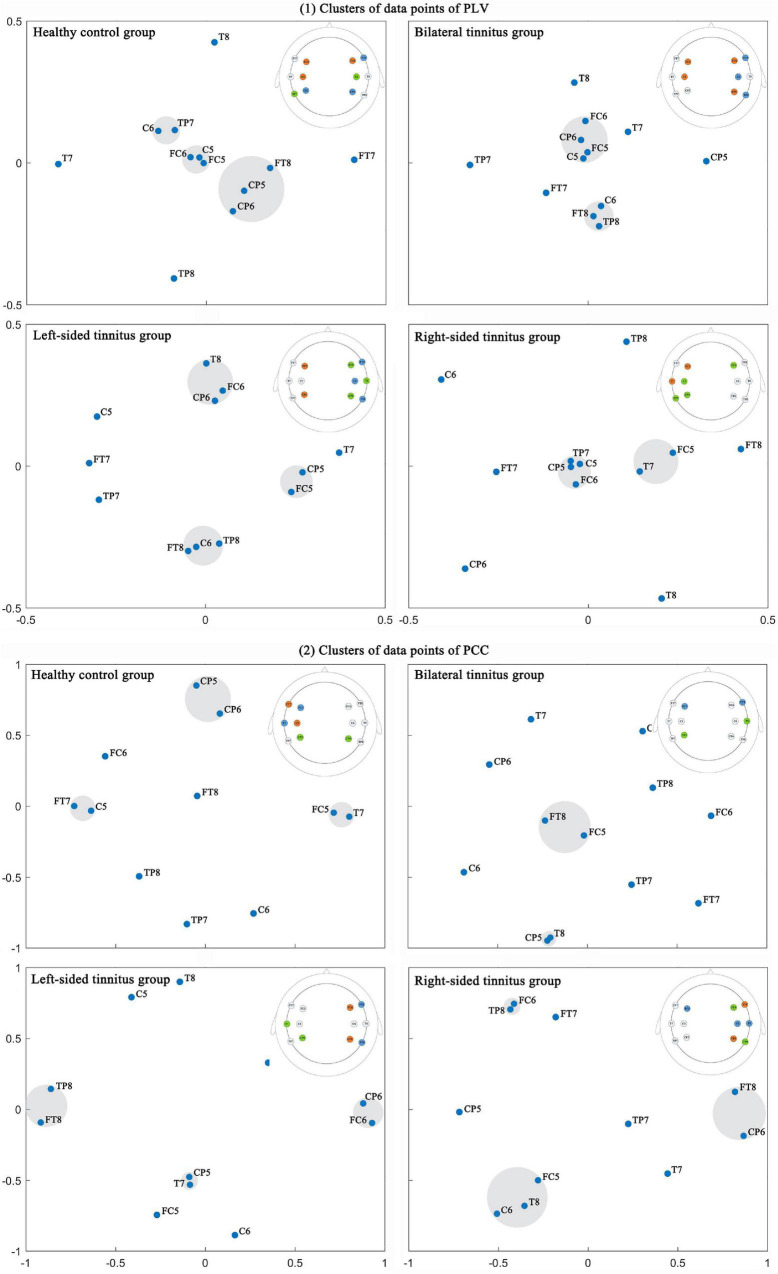
The data point clusters of multidimensional scaling of connectivity feature value were obtained by multidimensional cluster statistics. The highlighted area was a cluster of data points. The channels in the same cluster were highlighted with the same color in the brain topographic map, and which is in the upper right area of each picture. (1) Clusters of data points of PLV; (2) Clusters of data points of PCC.

We use the method of multidimensional cluster statistics (MCS) to analyze the connectivity feature value from another perspective, the MCS allows for a comparison of clusters of data points in multidimensional space, and which can quantify the similarities and dissimilarities of cortical activation patterns across recording channels (Krauss et al., 2018). First, multidimensional scaling (MDS) is used to reduce the dimension of the connection matrix (Burt and Torgerson, 1959; Kruskal, 1964a,b), then we visualize the data, and we calculate the Euclidean distance between points (channels) and perform clusters division finally (Figure 7). First, we look at the cluster analysis of the PLV. There are three similar connectivity activations (SCA) between the channels on both sides of the brain in the healthy control group. Compared with the healthy control group, the SCA between the channels on both sides of the brain is reduced to one in the bilateral tinnitus group, and there is one SCA on the right side of the brain at the same time. In the left-sided tinnitus group, there are two SCA on the right side of the brain, and there is also one SCA on the left side of the brain. In the right-sided tinnitus group, there are two SCA on the left side of the brain. Although it contains one channel on the right side of the brain, most of the similar channels are located on the left side of the brain. Then, we look at the cluster analysis of the PCC. There are two SCA on the left side of the brain and one SCA between both sides of the brain in the healthy control group; The binaural tinnitus group also has two SCA between both sides of the brain, and the similarity is stronger, compared to the healthy control group, the SCA on the left side of the brain disappears. Compared with the healthy control group, the left-sided tinnitus group has two new SCA on the right side of the brain, but the SCA between both sides of the brain disappears. Compared with the left-sided tinnitus group, the right-sided tinnitus group has stronger SCA on the right side of the brain, and the SCA between both sides of the brain also appears.

## Discussion

Two cross-validation methods were selected to match the SVM algorithm model which greatly improve the classification accuracy of the SVM classifier, but they also increased the calculation time, especially the Loo-CV. The SVM model with the Loo-CV always used almost 100% data in each training. It was very effective for small sample classification problems, but when the sample size became larger (for example, this experiment had 2,300 data samples), the calculation time of the model would be greatly increased, which also meant that the model was not suitable for real-time diagnostic evaluation. Compared with SVM, the classification efficiency of MLP was higher, mainly because the core solution of the classification model was different. The former was to find the global optimal solution, while the latter was the local optimal solution, so the calculation speed of the MLP was faster. At the same time, the classification accuracy of MLP was also close to SVM, which was more suitable for real-time diagnostic evaluation. Similarly, the CNN model usually performed multi-layer pooling of data, the computational efficiency was not too high, and the running time was close to the worst SVM-LooCV. The statistical results also showed that the classification accuracy obtained by different methods had significant differences (*p* < 0.01). In other words, the four machine learning models had significant differences. And MLP model performed excellently in the four model algorithms in terms of classification accuracy and computing time.

The classification accuracy of PCC or PLV features in the four classification models was better than other features, which verified that the functional connectivity feature, especially PCC or PLV, was more suitable as a biomarker of tinnitus. However, the result of the PCC+PLV was not better than the accuracy of a single feature, and there was a reduction. In the future, we should adopt a more effective combination mechanism to improve the classification accuracy, for example, extracting the more relevant parts of the single feature for combination. In the left-sided tinnitus group, there is a significant difference in the classification accuracy of LFT patients and HFT patients, but the difference in the recognition accuracy of the two is very small. And it will not affect the diagnosis of tinnitus location. In our research on the identification of tinnitus location, the difference between LFT and HFT patients can be ignored.

A recent study on the neural connection of tinnitus recruited a group of special tinnitus patients who can actively turn on the state of tinnitus through physical stimulation or sound stimulation (Zhang J. et al., 2021). The study noted that the occurrence of unilateral tinnitus would have an effect on the EEG signals in the contralateral brain area by locating the EEG signals source, and the symptoms of bilateral tinnitus mainly affected the EEG signals in the left side of the brain. Still, this conclusion might have individual differences because the data was only from one participant. Some research supported that the changes in the EEG signals in the left auditory area were related to the formation of tinnitus and did not distinguish the effects of unilateral and bilateral tinnitus (Ashton et al., 2007; Schecklmann et al., 2013).

In this study, the cluster analysis of the PLV and PCC showed that bilateral tinnitus mainly affected the connectivity of the auditory cortex on both sides of the brain and the connectivity of the right or left auditory cortex. The left-sided tinnitus mainly affected the connectivity of the right auditory cortex. And the right-sided tinnitus affected the connectivity of the left auditory cortex, and it also affected the connectivity of the right auditory cortex. An interesting finding was that tinnitus patients always had increased causal connectivity on the right side of the brain (Cai et al., 2020).

As far as we know, there is no previous study on the objective classification of tinnitus location through neural network algorithms, and this study also confirms that functional connectivity features, especially PCC or PLV, are more efficient in identifying the location of tinnitus. This study provides evidence for the effectiveness of the functional connectivity features of resting-state EEG as biomarkers for tinnitus.

In addition, an epidemiologic perspective on tinnitus pointed out that the prevalence of tinnitus among military veterans was relatively high. In a tinnitus screening survey for veterans, the prevalence rate reached 63%, and the participants were young veterans with an average age of 34.8, who had been out of the army for less than 3 years. A more noteworthy problem was that only approximately 20% of them would seek clinical intervention, and most of them respected tinnitus as being “not a problem” or “a small problem” (Henry et al., 2020). If we realize the rapid detection and objective diagnosis of tinnitus, it will greatly improve the life happiness index of military veterans, and this is especially true for the general population.

This study also has certain limitations. Considering that we want to carry out the objective diagnosis of tinnitus more conveniently in the future, we utilize 12-channel EEG on the auditory cortex on both sides of the brain for analytical research, because the 12-channel EEG acquisition experiment is simpler and faster than 64-channel. Although the 12-channel EEG signal can show obvious differences, the connectivity of the auditory regions on both sides of the brain cannot fully represent the functional network connectivity of the whole brain. In addition, the audiogram measured in this study is 8 khz. Although extending to higher frequencies above 8 khz may improve our ability to detect invisible hearing loss, the clinical significance of the high-frequency hearing test is still unclear (Balatsouras et al., 2005; Schmuziger et al., 2007). Finally, our work only researches tinnitus patients without hearing loss, but it is necessary to conduct further experiments on tinnitus patients with hearing loss to confirm the superiority of connectivity features in distinguishing tinnitus patients from healthy people.

## Conclusion

This study showed that connectivity features, especially PLV and PCC, could be biomarkers of tinnitus location in the resting-state EEG signals. Classification accuracy was highest when the SVM-10CV algorithm or the MLP algorithm was applied to the PCC feature sets, achieving final average classification accuracies of 99.42 and 99.1%, respectively. And based on the PLV feature, the classification result was also particularly good. Together, these results confirmed the feasibility of this method and the method could also meet the needs of objective diagnosis of tinnitus location.

## Data Availability Statement

The datasets presented in this study can be found in online repositories. The names of the repository/repositories and accession number(s) can be found: https://pan.baidu.com/s/1MOw__KoFKZQWpRVzA7_6WQ, password:jhl6.

## Ethics Statement

The studies involving human participants were reviewed and approved by the Ethics Committee of Chinese PLA General Hospital. The patients/participants provided their written informed consent to participate in this study. Written informed consent was obtained from the individual(s) for the publication of any potentially identifiable images or data included in this article.

## Author Contributions

ZL: study design, data analysis, result interpretation, manuscript drafting, and revision. XW: study design, result interpretation, manuscript drafting, and revision. WS and SY: conceptualization and methodology. DZ: funding acquisition. JH and DW: supervision. JL and HX: data curation. YZ: visualization and investigation. PL: software and validation. BZ and HC: resources. YL and XL: experiment management. All authors had contributions on preparing this manuscript.

## Conflict of Interest

DZ was employed by the company BetterLifeMedical. The remaining authors declare that the research was conducted in the absence of any commercial or financial relationships that could be construed as a potential conflict of interest.

## Publisher’s Note

All claims expressed in this article are solely those of the authors and do not necessarily represent those of their affiliated organizations, or those of the publisher, the editors and the reviewers. Any product that may be evaluated in this article, or claim that may be made by its manufacturer, is not guaranteed or endorsed by the publisher.
